# How Can a Histidine Kinase Respond to Mechanical Stress?

**DOI:** 10.3389/fmicb.2021.655942

**Published:** 2021-07-15

**Authors:** Linda J. Kenney

**Affiliations:** Department of Biochemistry and Molecular Biology, University of Texas Medical Branch, Galveston, TX, United States

**Keywords:** mechanosignaling, lipid allostery, EnvZ, histidine kinase, nanodiscs, catch bonds, mechanosensitive channels, biofilms

## Abstract

Bacteria respond to physical forces perceived as mechanical stress as part of their comprehensive environmental sensing strategy. Histidine kinases can then funnel diverse environmental stimuli into changes in gene expression through a series of phosphorelay reactions. Because histidine kinases are most often embedded in the inner membrane, they can be sensitive to changes in membrane tension that occurs, for example, in response to osmotic stress, or when deformation of the cell body occurs upon encountering a surface before forming biofilms, or inside the host in response to shear stress in the kidney, intestine, lungs, or blood stream. A summary of our recent work that links the histidine kinase EnvZ to mechanical changes in the inner membrane is provided and placed in a context of other bacterial systems that respond to mechanical stress.

## Introduction

**Figure 1:**
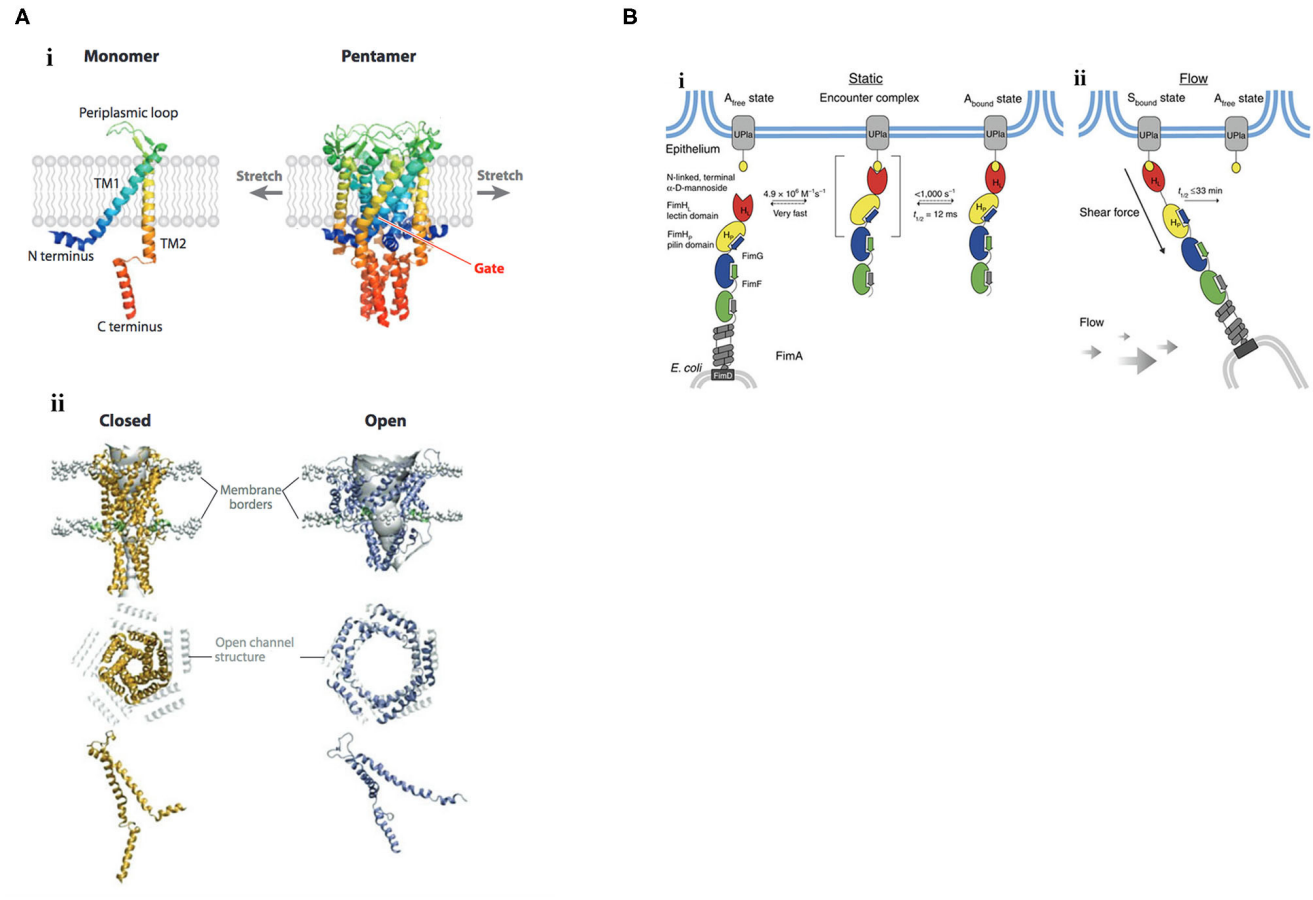
Mechanisms of mechanosignaling in bacteria. **(A)** Structure and gating mechanism of MscL. (i) X-ray structure of the MscL from *Mycobacterium tuberculosis*. The channel monomer (left) is composed of two α-helical TM domains, TM1 and TM2; cytoplasmic N- and C-terminal domains; and a central periplasmic loop. The five TM1 domains form a tightly packed bundle funneling to a hydrophobic constriction, functioning as the gate (right). (ii) Three-dimensional reconstruction of the pentameric MscL of *E. coli* viewed in the plane of the membrane (top) and from above (middle). The channel is shown in its closed (left) and open (right) states. In the top row, lipid phosphate headgroups are shown as gray balls to indicate the outer and inner borders of the membrane. The extent of rearrangement of a single MscL subunit from the closed to the open conformation is depicted at the bottom. Membrane tension flattens MscL in the direction of the pore axis due to an increased tilt in the TM1 and TM2 helices. Closed and open structures of MscL (middle) are superimposed on the open channel structure (depicted in gray). TM, transmembrane. Reprinted with permission from Kung et al. (2010). **(B)** Catch-bond mechanism of FimH-mediated cell adhesion. (i) In the absence of tensile mechanical force, formation of the FimH-Uroplakin 1a (UPIa) complex comprises the highly dynamic transition of the **A**_**free**_ to the **A**_**bound**_ state. Dissociation of the receptor from the FimH lectin domain in the **A**_**bound**_ state is promoted via dynamic allostery by the pilin domain that acts as a negative allosteric regulator. (ii) Shear force increases the population of the **S**_**bound**_ state of FimH, in which the pilin and lectin domains are separated. The dissociation of **S**_**bound**_ under shear force is slowed down up to 100,000-fold compared with **A**_**bound**_. The indicated rate constants and half-lives correspond to the interaction between FimH^F18^ and the model ligand HM. Rate limiting reactions are indicated by solid arrows, and fast, non-limiting reactions by dashed arrows. Reprinted with permission from Sauer et al. (2016).

Bacteria are sensitive to physical forces and experience them as part of their environmental sensing strategies, for example during growth and elongation, cell division or cell envelope remodeling, or as they adhere to a surface in order to form biofilms, and as they experience shear stress during colonization and infection as pathogens [see Harper and Hernandez (2020) for a recent review]. Although bacteria have long been known to be sensitive to their mechanical environment (Koch et al., 1982), understanding the effects of physical forces on bacterial physiology has been limited by their small size (~ 1 μm). Herein, I first summarize some well-characterized examples of force effects on bacterial systems (Figure 1) and then describe some recent advances in understanding the effects of membrane tension and lipids on bacterial two-component signaling systems, with an emphasis on the EnvZ/OmpR two-component signaling system.

## Bilayer-Mediated Gating of Mechanosensitive Channels

Effects of membrane tension are probably best described on mechanosensitive channels, in which the bilayer mediates channel gating. The major mechanosensitive channels MscS, MscL protect bacteria from hypoosmotic shock [see Kung et al. (2010) for a review]. When cells experience a hypoosmotic downshift, mechanosensitive channels act as emergency valves to release cytoplasmic solutes to enable a rapid decrease in osmolality (Levina et al., 1999). Mechanosensitive channels directly interact with the membrane phospholipids (Nomura et al., 2012), and are gated in response to changes in membrane tension. Reconstitution of purified channel proteins into liposomes of varying lipid composition enabled the analysis of mechanosensitive channels by patch-clamp electrophysiology (Nomura et al., 2012). Membranes that were thicker, by being composed of phospholipids having longer acyl chain lengths (Perozo et al., 2002), or containing cholesterol (Nomura et al., 2012), raised the activation threshold, whereas thinner membranes composed of phospholipids of shorter acyl chain lengths lowered the activation barrier (Perozo et al., 2002). An N-terminal amphipathic helix of MscL acts as a crucial structural element by stabilizing the closed state and coupling the channel to the membrane (Bavi et al., 2016). This horizontal helix directly links membrane bilayer fluctuations to protein conformation (Iscla et al., 2008), exquisitely coupling membrane dynamics to channel open or closed states (see Figure 1A), as first suggested by Segrest et al. (1974).

## Protein Allostery Mediates Catch Bond Strength

Catch bonds play a significant role in bacterial adhesion, especially during transit of *E. coli* in the intestinal tract or uropathogenic *E. coli* in the renal tubules, where firm adhesion of bacteria in the face of hydrodynamic flow is required. Mechanosensitive catch bonds are a non-covalent bond whose dissociation constant increases upon application of tensile force. The concept of a catch bond is similar to a Chinese finger trap, where the bond strengthens when force is applied and weakens when force is released (Thomas, 2009). The bacterial adhesion FimH is a two-domain protein at the tip of Type I pili that recognizes terminal mannoses on epithelial glycoproteins. In the absence of tensile force, the FimH pilin domain allosterically accelerates (by 100,000-fold) spontaneous ligand dissociation from the FimH lectin domain, resulting in weak affinity (Sauer et al., 2016). Mechanical stress physically separates the FimH domains and abolishes the interdomain allostery, and the affinity of FimH for the lectin is increased (see Figure 1B). Thus, protein allostery contributes to mechanotransduction in catch bonds by altering bond strength.

Signal transduction in bacteria is largely perceived by two-component signaling systems that respond to environmental stress coupled to a phosphorelay that usually results in changes in gene expression. Thus, it seems logical that two-component systems would also be sensitive to changes in membrane tension. In the remaining sections, we focus on what is currently known regarding mechanosignaling in gram-negative bacterial two-component systems.

## Mechanical Effects on Two Component Signaling Systems

Bacteria sense and respond to their environment largely through the use of two-component regulatory systems that function as a histidine kinase (HK)-response regulator (RR) pair. The HK is typically an inner membrane protein and the RR is most often a cytoplasmic two-domain DNA binding protein [see Hoch and Silhavy (1995) for numerous examples]. The two components function in a phosphorelay that involves ATP binding by the HK and autophosphorylation on a conserved histidine residue. The phosphoryl group is subsequently transferred to a conserved aspartic acid in the N-terminal phosphorylation domain of the RR. Phosphorylation in the N-terminus of the RR stimulates dimerization and communicates with the C-terminus for enhanced DNA binding and transcriptional activation. In *E. coli*, the EnvZ/OmpR two-component system functions to regulate many genes in response to acid and osmotic stress (Chakraborty et al., 2017; Chakraborty and Kenney, 2018), including the differential expression of outer membrane porins OmpF and OmpC [reviewed in Kenney and Anand (2020)]. In *Salmonella enterica* serovars Typhi and Typhimurium, EnvZ/OmpR is stimulated by acid stress (Chakraborty et al., 2015; Kenney, 2019) to up-regulate the SsrA/B two-component system (Liew et al., 2019) required for intracellular survival and virulence (Lee et al., 2000; Feng et al., 2003).

**Figure 2:**
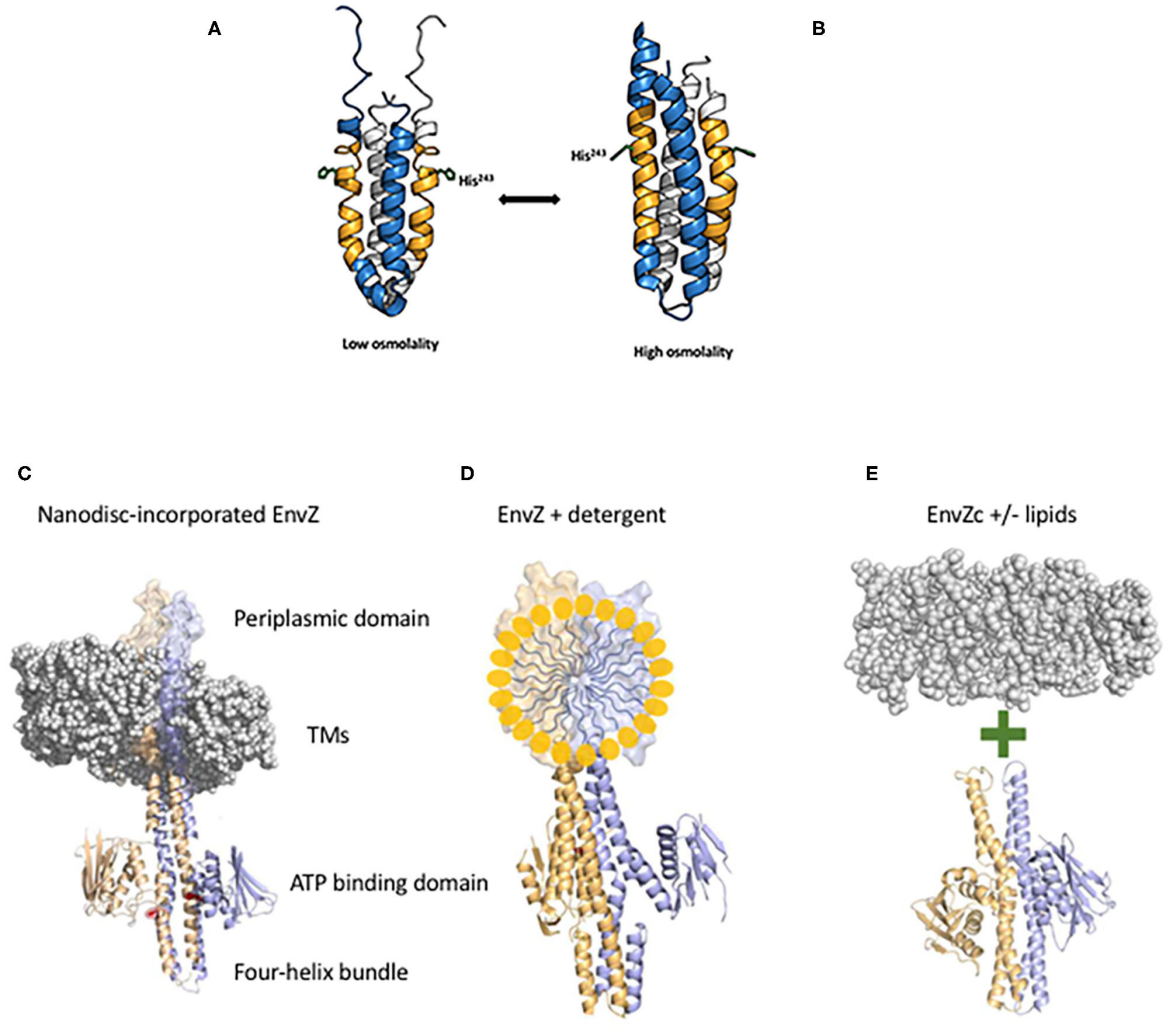
EnvZc four-helix bundle subdomain structure at low osmolality **(A)** and at high osmolality **(B)**. The low osmolality structure was composed of residues 223–289 and was solved by NMR (PDB ID:1JOY) (Tanaka et al., 1998), the high osmolality structure was a chimera of EnvZ with the *Archaeoglobus fulgidus* Af1503 HAMP domain (PDB ID:4CTI) (Ferris et al., 2014). The amber peptide was identified from HDXMS analysis highlighting the change from poorly ordered to highly ordered during the switch from low to high osmolality (Wang et al., 2012). The sidechain of the histidine that is phosphorylated (His^243^) is shown in stick form. At low osmolality, His^243^ is positioned by a coordinated relay of interactions from the backbone carbonyl of Ala^239^ (one helical turn above His^243)^ through the imidazole side chain of His^243^ to the carboxylic acid side chain of Asp^244^. Intrinsic disorder within the amber peptide affords a low basal level of autophosphorylation. High osmolality stabilizes the helical backbone and relieves the Ala^239^ carbonyl link to anchor the His^243^ imidazole by a H-bond with Asp^244^ at the Nδ of His^243^. This enables phosphotransfer to the Nε of His^243^ [see Kenney and Anand (2020) for more details]. Thus, the core of osmosensing is a His-Asp/Glu dyad positioned within a flexible helix. Various EnvZ constructs employed in the study. **(C)** The EnvZ periplasmic domain protrudes above the membrane (shown as a space filling model PDB ID:3J00, the EnvZ dimer is from PDB ID:4CTI). The transmembrane domains (TMs) connect to the four-helix bundle formed from a dimer of two monomers shown in lavender and pale orange. A single His^243^ side chain is highlighted in red (phosphorylation site) and the ATP binding domains flank His^243^ [from Kenney and Anand (2020)]. **(D)** Full-length EnvZ solubilized in detergent micelles for comparison with nanodiscs and *E. coli* lipids. **(E)** The activity of EnvZc alone (bottom) was compared with EnvZc in the presence of lipids (see Table 1).

HKs are typically organized with their N-and C-termini in the cytoplasm, with two transmembrane domains, a periplasmic loop and a large C-terminal portion that is subdivided into a four-helix bundle containing the phosphorylated histidine and an ATP binding domain [see Kenney and Anand (2020)]. In general, HKs are much less abundant compared to their cognate RRs (Cai and Inouye, 2002; Liew et al., 2019). The low copy number of the HKs coupled with their usual location in the inner membrane makes studying their activation mechanisms difficult. To circumvent this problem, we initially explored the protein dynamics of the C-terminus that was liberated from the membrane (EnvZc, comprising residues 180–450) using amide hydrogen deuterium exchange mass spectrometry (HDXMS) (Wang et al., 2012). A 17-amino acid peptide flanking the phosphorylatable histidine was exquisitely sensitive to osmolytes and defined a cytoplasmic locus for osmosensing (see Figure 2A). At low osmolality, the peptide containing the phosphorylatable histidine had higher rates of exchange and the helix was locally disordered. The presence of osmolytes reduced exchange and promoted helical stabilization through the addition of several H-bonds (Figure 2B; Wang et al., 2012). These events increased autophosphorylation and subsequent OmpR activation. It was surprising that EnvZc, which was no longer located in the inner membrane, was still capable of osmosensing, as reported by the upregulation of an *ompC-lacZ* transcriptional fusion at high osmolality. This finding raised the question then as to what role (if any) does the membrane and the transmembrane domains of EnvZ play in controlling EnvZ activity?

The answer came in part from the use of purified, full-length detergent-solubilized EnvZ protein that was embedded into phospholipid bilayer nanodiscs (Figure 2C; Ghosh et al., 2017). Nanodiscs were useful for this purpose, because they are monodisperse, exhibit long term stability compared to proteoliposomes, and have a wide range of membrane protein applications (Nath et al., 2007). Although the use of nanodiscs eliminated the sidedness compared to the native protein *in situ*, their use enabled us to manipulate the membrane lipid composition, and to examine the effect of membrane lipids in regulating EnvZ receptor function through an interaction with the transmembrane segments (TMs) or the HAMP domain (Figure 2D), which were lacking in the EnvZc construct (Wang et al., 2012). With every construct that we explored, phosphorylation of EnvZ was stimulated in response to increasing osmolality by about 4-fold. Surprisingly, however, the addition of *E. coli* lipids to EnvZc (Figure 2E) dramatically increased ATP turnover at both low and high osmolality (25- or 14-fold, respectively), compared to EnvZc alone (see Table 1). A similar level of stimulation of ATP turnover (14- to 15-fold) was also observed with the full-length EnvZ protein embedded into nanodiscs, indicating that membrane lipid effects on the ATP binding domain was indeed the manifestation of membrane tension on EnvZ.

**Table 1:** Lipid interactions enhance EnvZ autophosphorylation [adapted from Ghosh et al. (2017)].

**Fold-Stimulation compared to EnvZc**	**ADP produced (Mole/Min per Mole of EnvZ)**	
	**Low osmolality**	**High osmolality**
EnvZc + *E. coli* lipids	24	14
EnvZ + *E. coli* lipid nanodiscs	14	15

*EnvZ autophosphorylation was measured using an in vitro ADP Glo kinase assay (Promega, Madison WI), which uses luminescence to quantify the amount of ADP produced over time. Measurements were performed at low osmolality (100 mOsm/kg) and high osmolality (780 mOsm/kg). The activity of EnvZc alone was 0.09 mole ADP/mol EnvZc per minute at low osmolality and 0.32 mole ADP/mol EnvZc per minute at high osmolality. See Ghosh et al. (2017) for more details*.

The subsequent application of HDXMS enabled us to determine which region of EnvZ was susceptible to lipids. The highly conserved glycine-rich motif of the ATP binding domain showed increased exchange in the presence of lipids with both EnvZc and with full-length EnvZ, exposing a previously unobserved effect of lipids on an HK. We speculated that as cells reduce their volume when they encounter a high osmotic environment, mechanical effects on the membrane lipids would exert an effect on the ATP binding domain of EnvZ, stimulating an increase in EnvZ autophosphorylation. Thus, lipids exert allosteric effects mediated through the ATP binding domain that drive HK signaling in response to osmotic stress.

Local anesthetics such as procaine and phenylethyl alcohol (PEA) partition into the inner membrane, yet they lead to a reduction in the level of OmpF and an increase in OmpC in the outer membrane of *E. coli*, mimicking a high osmolality phenotype (Granett and Villarejo, 1982; Pages and Lazdunski, 1982). In the case of procaine, its action was reported to be dependent upon EnvZ (Granett and Villarejo, 1982; Taylor et al., 1983). Until recently, it was not possible to directly connect lipid effects to HK signaling. The observation that membrane lipids altered ATP turnover of EnvZ and stimulated phosphorylation (Ghosh et al., 2017) provided an explanation for how local anesthetics such as procaine (Granett and Villarejo, 1982) and other membrane perturbants (Rampersaud and Inouye, 1991) affect EnvZ signaling. By altering the lipids (Papahadjopoulos, 1972), these agents can exert an indirect effect on the glycine-rich loop of the ATP binding domain, stimulating ATP turnover and subsequent autophosphorylation of EnvZ at His^243^. Increasing EnvZ autophosphorylation subsequently increases OmpR~P levels, driving repression of *ompF* and activation of *ompC* transcription (Granett and Villarejo, 1981, 1982; Rampersaud et al., 1994). Thus, modification of the lipid bilayer by local anesthetics can be understood in terms of direct mechanical effect of lipids on the ATP binding domain of EnvZ.

**Figure 3:**
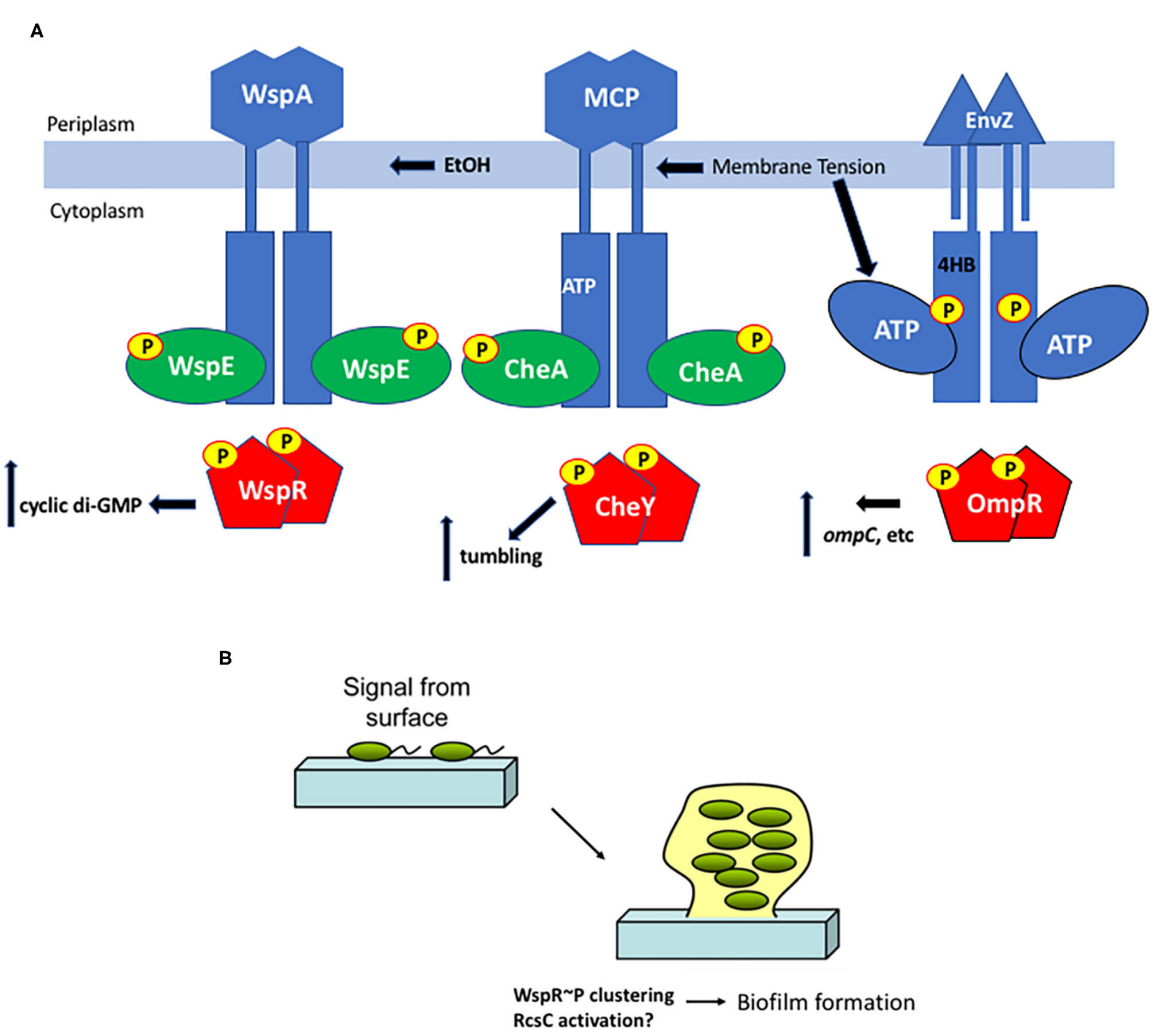
Surface sensing in bacteria. **(A)** Membrane tension from osmotic stress, altered lipids or EtOH produced from a co-infection with *C. albicans* alters the chemoreceptors WspA and MCPs and/or the EnvZ HK directly and stimulates ATP turnover, increasing RR phosphorylation in turn, resulting in a change in output, depending on the RR. **(B)** Interaction with a surface is an early step in biofilm formation. Cells transition from the motile, planktonic state to interact with a surface via pili or other appendages and then begin the biofilm process. Deformation of the bacterial cell as it encounters a surface could then drive signaling steps that eventually lead to aggregation and production of matrix proteins.

To obtain a comprehensive understanding of downstream signaling events, a comparison with the much-studied chemotaxis system is informative. Although the bacterial chemotaxis system is an atypical “two-component” system, the common features include the HK CheA and the single domain RR CheY. Significant differences include the cytoplasmic location of the kinase CheA (Stock et al., 1989), although gram-positive organisms have soluble, cytoplasmic HKs as well [described in Hoch and Silhavy (1995)], and the histidine that is phosphorylated is in a different location (the P1 domain) from the more classical membrane-bound kinases (Hess et al., 1988). Changes in membrane tension resulting from osmotic shock act as a repellant by mechanically perturbing chemoreceptors, driving enhanced activity of the CheA kinase (Vaknin and Berg, 2006). The CheA kinase then phosphorylates the CheY RR to switch the direction of rotation of the flagellar motor from counterclockwise (smooth swimming), to clockwise (tumbling). In this example, the chemoreceptors act as a mediator between membrane tension and the soluble kinase CheA. Thus, both EnvZ and CheA are activated by membrane tension (compared in Figure 3A).

Similarly, a role of the ATP binding domain in HK signaling has been described in both the EnvZ/OmpR system (see above) and the chemotaxis system. ATP binding orders the “ATP lid” and creates a binding site for P1, the domain that contains the phosphorylated histidyl residue of CheA (Jun et al., 2020). Similarly, membrane lipid interactions with the ATP binding domain of EnvZ increase autophosphorylation of His^243^. EnvZc is as sensitive to lipids as the full-length EnvZ, suggesting that it may localize in proximity to the inner membrane (Ghosh et al., 2017). Thus, at least in these two systems, kinase control by environmental stimuli appears to be exclusively at the level of autophosphorylation and is sensitive to membrane tension.

## Mechanical Stress/Surface Sensing

During biofilm formation, it is believed that sessile bacteria first interact with a surface via appendages such as pili and flagella (Zhang and Normark, 1996; Otto et al., 2001; Bhomkar et al., 2010; Lele et al., 2013; Tipping et al., 2013; Lee and Belas, 2015), and then eventually the bacterial cell body encounters the surface. Physical contact between the surface and the cell body is presumably sensed as envelope stress that leads to downstream changes in gene expression (Figure 3). When planktonic, motile bacteria approach a surface, flagellar rotation decreases, presumably due to either a change in viscosity or through the physical interaction with the surface (McCarter et al., 1988). This virulence/biofilm lifestyle switch has been most thoroughly studied in *Pseudomonas aeruginosa*. A complete proteomic analysis was obtained from *P. aeruginosa* immobilized on glass wool comparing attached and unattached populations. Six hundred sixteen proteins showed modified abundance, including several two-component systems (Crouzet et al., 2017). Two-component systems that are intricately involved in the sessile-motile switch and may be good candidates for surface sensors include the chemosensory signaling system Wsp (Huangyutitham et al., 2013) in *P. aeruginosa* and the RcsCDB system in *E. coli* (Ferrieres and Clarke, 2003).

In the Wsp chemosensory signaling system, the membrane bound receptor WspA responds to surface signals and activates the soluble, cytoplasmic HK WspE, which in turn phosphorylates the hybrid RR WspR. Instead of coupling phosphorylation in the N-terminal receiver domain to a DNA binding domain in the C-terminus, WspR contains a guanylate cyclase domain that produces cyclic di-GMP, stimulating biofilm formation. When *P. aeruginosa* encounters a surface, phospho-WspR forms clusters in the cytoplasm that stimulate the cyclase activity (Huangyutitham et al., 2013). WspA localizes laterally along the cell, and the periplasmic and transmembrane domains of WspA are not essential for surface sensing, although the system performed better when they were present (O'Connor et al., 2012). Additional components do not appear to be required, since surface sensing could be reconstituted in *E. coli* (M. R. Parsek and C. S. Harwood, personal communication). More recently, it was reported that ethanol, produced during co-infections of *C. albicans* and *P. aeruginosa*, stimulated biofilm production in a process requiring WspR and the chemosensory receptor WspA (Chen et al., 2014). The authors proposed that ethanol and other alcohols can increase the rigidity of cell membranes by promoting an altered composition of fatty acids (Ingram and Buttke, 1984). It will now be worthwhile to determine whether the membrane-localized WspA can be activated by changes in lipid composition or physical changes in *P. aeruginosa* membranes (see Figure 3A). Perhaps even more interesting was the observation that cell surface-sensing resulted in a heterogeneous population of low cyclic di-GMP cells and high cyclic di-GMP cells that perform complementary tasks in the early stages of biofilm production (Armbruster et al., 2019). The Wsp chemosensory system was essential for establishing the heterogeneity that then creates a division of labor between cells involved in surface exploration and cells involved in polysaccharide production.

In *E. coli*, bacterial signaling systems that were reported to be involved in surface sensing are the RcsCDB phosphorelay and the CpxA/R system. The sensor kinase RcsC plays an important role in controlling the remodeling of the *E. coli* surface in response to growth on a solid surface and during biofilm formation (Ferrieres and Clarke, 2003). The Cpx two-component signal transduction pathway responds specifically to stress caused by disturbances in the cell envelope and CpxA then activates CpxR to express genes encoding periplasmic protein folding and degrading factors. The outer membrane lipoprotein NlpE was reported to discriminate between surface adhesion vs. the misfolded protein pathway in that it was essential for the activation of Cpx specifically during surface adhesion, but not in the response to misfolded proteins in the cell envelope (Otto and Silhavy, 2002). This finding was disputed by a more recent study that used single cell analysis of cells in a microfluidic device and reported that the RcsCDB system was activated upon surface attachment, but the CpxA/R system was not (Kimkes and Heinemann, 2018), confirming an earlier study that implicated RcsC in surface sensing (Ferrieres and Clarke, 2003). At the present time, it still remains a mystery as to how RcsC functions to sense surfaces to drive downstream activation of the biofilm pathway (Kimkes and Heinemann, 2020). Sensing may involve a disruption of the membrane lipoprotein RcsF and its interaction with outer membrane proteins (Konovalova et al., 2014).

## Concluding Remarks

Although much remains to be understood in molecular terms as to how bacteria respond to mechanical stress, it is clear that some progress has been achieved. Bacteria respond to changes in membrane tension via a combination of mechanical events, including both lipid allostery and protein allostery. In the examples of lipid allostery, direct interaction with membrane phospholipids leads to bilayer-mediated gating in the case of mechanosensitive channels, or to direct effects on ATP binding and subsequent HK phosphorylation in the case of EnvZ and CheA (and possibly WspA) (summarized in Figure 3A). Cell body deformation when bacteria encounter a surface is also sensed as “envelope stress”, activating RcsC in an unknown series of events and driving early steps in the biofilm pathway (Figure 3B). In the case of FimH-mediated catch bonds, protein allostery is abolished as force separates protein domains, and the catch bond is strengthened. New tools such as membrane tension sensors (Dal Molin et al., 2015; Soleimanpour et al., 2016; Colom et al., 2018), and new applications of existing methods including: gel encapsulation (Tuson et al., 2012), optical traps (Wang et al., 2010), microfluidic devices (Amir et al., 2014; Sun et al., 2014; Chang et al., 2018; Sanfilippo et al., 2019), atomic force microscopy (Yao et al., 1999; Deng et al., 2011; Mularski et al., 2015), and the ability to study single cells within a population will provide useful tools in defining additional mechanisms of mechanotransduction in bacteria.

## Data Availability Statement

The original contributions presented in the study are included in the article/supplementary material, further inquiries can be directed to the corresponding author/s.

## Author Contributions

The author confirms being the sole contributor of this work and has approved it for publication.

## Conflict of Interest

The author declares that the research was conducted in the absence of any commercial or financial relationships that could be construed as a potential conflict of interest.

## Abbreviations

HK: histidine kinase
RR: response regulator
HDXMS: amide hydrogen deuterium exchange mass spectrometry.
